# Half the Chromosome It Used to Be: Identifying Cancer Treatments Targeting Aneuploid Losses

**DOI:** 10.3390/genes16060708

**Published:** 2025-06-14

**Authors:** Andrew O. Disharoon, Joe R. Delaney

**Affiliations:** Department of Biochemistry and Molecular Biology, Medical University of South Carolina, Charleston, SC 29425, USA; disharoa@musc.edu

**Keywords:** oncology, cancer, chemotherapy, precision oncology, aneuploidy

## Abstract

Background/Objectives: Aneuploidy is near-ubiquitous in cancer and can decrease chemotherapy efficacy while also sensitizing cells to other drugs. Methods: To systematically identify treatment strategies that target aneuploid cancers, data were integrated from The Cancer Genome Atlas (TCGA; 10,967 samples, 16,948 aneuploidy events) and the Broad Institute’s Profiling Relative Inhibition Simultaneously in Mixtures (PRISM) screen of 578 cancer cell lines and 4518 compounds. Results: Our analyses uncovered 37,720 significant positive and negative associations linking specific aneuploidies and treatments with patient prognosis or cell viability. Within TCGA data, 22 treatments correlated with improved 5-year survival for specific aneuploid cancers, whereas 46 were linked to worse outcomes. A complementary analysis of PRISM identified 17,946 compound–aneuploidy associations and 16,189 mechanism of action (MOA)–aneuploidy associations. Pathway-altering compounds that selectively reduce viability in cells with aneuploidy profiles were discovered, including an unexpectedly prominent number of glucocorticoid receptor agonists. Conclusions: This integrated dataset provides a resource for designing therapeutic decision hypotheses, identifying drug-repurposing opportunities, and informing future studies aimed at targeting aneuploidy-induced vulnerabilities in cancer.

## 1. Introduction

Cancer is largely a consequence of genomic dysregulation made pathogenic through successful evasion, expansion, and exploitation of its parent-host. While not exclusively the driver of oncogenesis [1], this genomic dysregulation arises from a range of factors, including single-point mutations and large-scale chromosomal abnormalities such as aneuploidy. Aneuploidy is the abnormal number of chromosomes or chromosome arms, differing from the normal diploid (euploid) state [2,3]. Aneuploidy includes both losses and gains of chromosomal material and is most often a change of a single allele, or monoallelic, in tumors. Aneuploidy is distinct from polyploidy, the gain or amplification of entire genomes across all chromosome arms, which can precede further aneuploid variation. Aneuploidy’s genesis as an instigator or consequence of cancer evolution is well documented [2,4,5,6]. The majority of cancers either display or have the potential to display some sort of aneuploidy [7]. For example, 17p monoallelic loss and subsequent loss of wild-type *TP53*, a tumor suppressor gene, is a major driver of lung, breast, ovarian, leukemia, colon, esophagogastric, and other cancers [8]. Zygosity is important for tumor suppressor genes. Loss of a wild-type tumor suppressor allele with retention of mutated allele is a frequent driving event for oncogenesis [9]. Aneuploidy incurs the accumulation of moderate-impact genetic drivers of cancer through haploinsufficiency or triploproficiency [10,11]. Importantly, the prognosis of patients with higher aneuploidy scores (the total number of chromosomes with aneuploidy and polyploidy) is lower [3,12].

While aneuploidy frequently enhances chemotherapeutic resistance and immune evasion, it correlates with reduced cancer cellular proliferation [12,13,14,15]. Notably, some aneuploidies are rarely observed, suggesting that certain abnormalities reduce fitness or are lethal and thus selectively eliminated [3,16,17]. Curable cancers, including teratomas, papillary thyroid carcinomas, and choriocarcinomas, can have minimal to no point mutations or aneuploidy events [18,19]. Despite the fitness costs, the prevalence of specific aneuploidies in cancer may reflect a form of survivorship bias, where only those that confer an advantage propagate. The selection for cellular fitness of aneuploid cells may stem from haploinsufficient gene expression [10], mitotic segregation challenges [2], and increased immune visibility [20]. Yet, among the survivors, aneuploidy is very common, contributing to genomic and chromosomal instability (CIN), further driving cancer evolution [21,22,23]. By providing phenotypic flexibility, aneuploidy- and polyploidy-induced CIN enable tumors to adapt to their environment and resist chemotherapy [13]. Further, CIN can instigate or perpetuate aneuploidy [6]. Approximately 90% of solid tumors diverge from the euploid state [15], and while both solid and hematologic cancers can acquire additional chromosome arms, solid tumors are more likely to lose them [24]. This pattern may help explain the greater therapeutic recalcitrance of advanced solid tumors [25]. Higher aneuploidy scores (and, by extension, CIN) correlate with poorer patient survival, as these scores are often associated with more advanced cancers harboring multiple genomic-regulation failures [3,26]. Although general aneuploidy-driven CIN influences overall tumor biology, specific copy-number alteration (CNA) events also shape cancer characteristics. Notable amplifications (e.g., *ERRB2*) and biallelic losses (e.g., *BRCA1/2*) guide targeted treatment strategies, but these focal CNA events are relatively rare compared to widespread monoallelic changes that affect large swaths of the cancer genome [5,27,28,29]. For instance, the haploinsufficient expression of *TP53*, *BECN1*, and *PTEN* can foster oncogenesis, enhance metastasis, and compromise immune regulation [8,30,31,32,33]. Thus, while aneuploidy imposes fitness costs on cancer cells, its capacity to enable tumor adaptation and treatment resistance is a positive selection pressure. Conversely, aneuploidy creates both general and specific treatment opportunities. This juxtaposition highlights aneuploidy as a cellular burden yet a driver of cancer evolution.

Aneuploidy is largely distinct to cancer cells in the human body. The unique vulnerabilities of aneuploid cancers can be exploited by specific therapeutics to advance precision oncology. The haploinsufficient expression of tumor suppressors fuels oncogenesis; therefore, treatments that restore these gene functions [34], suppress haploid oncogenes, or push CIN beyond tolerable thresholds could represent a new generation of anti-aneuploid chemotherapeutics. Established compounds like PRIMA-1 [35], imatinib [36], and cisplatin [37] already demonstrate the potential to exert such effects. However, existing therapies aimed at destabilizing mitosis, a documented vulnerability inherent in aneuploid cells, show widely varying degrees of success in aneuploid cancer cells [37]. Moreover, the mitotic complications characteristic of aneuploidy can slow replication, diminishing the efficacy of treatments that target rapidly dividing cells [12]. This reduced efficacy underscores a shortcoming of current therapeutic strategies: the need to address the unique biology of aneuploid tumors, which often resist traditional cancer treatments. So, while the incorporation of tumor genotyping has improved patient outcomes [38], aneuploidy remains linked to poorer survival [12]. A better understanding of how aneuploidy interacts with existing and emerging chemotherapies is essential for advancing precision oncology. Nearly every tumor harbors multiple aneuploid events. Yet, no monoallelic changes are currently used as prognostic biomarkers. Incorporating aneuploidy data into clinical decision-making could therefore improve treatment strategies and patient care.

In this study, current and possible future treatments were evaluated regarding how they are associated with greater and reduced cancer cytotoxicity in aneuploid events. To determine how the current standard-of-care chemotherapeutics affect the prognosis of recipients with aneuploidy events, The Cancer Genome Atlas’ (TCGA) PanCancer Atlas studies were leveraged, containing 10,967 cancer samples with 16,948 aneuploidy events [39,40]. Potential future therapeutics targeting aneuploid loss were then identified by using the in vitro drug sensitivity screen published by the Broad Institute’s Profiling Relative Inhibition Simultaneously in Mixtures (PRISM) lab [41]. In this PRISM study, the viability of 578 cancer cell lines was measured when treated with 4518 compounds, many of which are non-oncology drugs with repurposing potential. The PRISM study has identified many possible repurposing compounds with cancer cytotoxicity and has been used as the basis for subsequent drug development and clinical translation. These findings were integrated with aneuploidy data from overlapping cell lines in the Cancer Cell Line Encyclopedia [42]. Using this combined dataset, compounds were identified that exhibit cancer cytotoxicity associated with specific aneuploid states.

## 2. Materials and Methods

### 2.1. Datasets

The most current information from the TCGA PanCancer Atlas studies [40] was obtained via API calls from cBioPortal [43]. These data were combined with data on putative arm level aneuploidy events for TCGA samples also found on cBioPortal. The aneuploidy status of these samples was calculated by GISTIC as described prior [39].

The Broad PRISM dataset was obtained from https://depmap.org/ [44]. The code used in this study can be found in the GitHub repository (https://github.com/jrdelaney/Disharoon_Delaney_Aneuploid_Selectivity_2025/, initiated 10 February 2025). Briefly, the calculations utilized Cancer Cell Line Encyclopedia “.seg” copy-number variant files [42], using a threshold of ±0.3 magnitude to call a copy number variant. An aneuploid event was called if 67% of the autosome chromosome arm was altered in one direction, the denominator (i.e., the length of the chromosome arm) as defined by the location of the first and last protein-coding gene on each chromosome arm. While this method captured many gain and loss events, it may underestimate alterations in certain specific chromosome arms, such as chromosome 17q.

### 2.2. TCGA Aneuploidy Normalization

The TCGA dataset does not have equal distribution of aneuploidy across each cancer type, nor equal representation of cancer types. To account for sampling biases influencing aneuploidy rates across the dataset, the proportion of samples representing each cancer type were calculated. These proportions were used as weights to adjust the observed aneuploidy rates, normalizing for overrepresented or underrepresented cancer types. This was calculated as follows:

$$P_{c}=\frac{N_{c}}{\sum_{c}N_{c}}$$

where *P_c_* is the proportion of the cancer type *c* in the dataset and *N_c_* is the number of samples for that cancer type.

For each chromosome arm, raw aneuploidy event counts were divided by these cancer-type proportions. This yielded a normalized rate for each chromosome arm across cancer types. The raw aneuploidy counts (*C_raw_*) for each chromosome arm and cancer type were adjusted by dividing by their respective proportions to derive normalized counts (*C_norm_*) as follows:

$$C_{norm}=\frac{C_{raw}}{P_{c}}$$

### 2.3. TCGA Survival Analyses

Using the TCGA datasets, a survival analysis was performed for each combination of either treatment (compound or MOA) and aneuploidy event (cancer types being aggregated together), or treatment, aneuploidy, and cancer type (un-aggregated). Patients with missing survival metrics, treatments, or chromosome arm data were excluded from the analysis for the specific combination of treatment, chromosome arm, and cancer type (if performing an un-aggregated analysis). Samples were assigned to either the “Loss” or “Other” (euploid and polyploid) aneuploidy group based on the aneuploidy status for each chromosome arm. Survival times (“Progress-Free Survival Months”) were paired with the censure criterion, disease-specific mortality status (“Disease-specific Survival status”). Analyses were not performed if either aneuploidy group contained fewer than 10 observations to ensure at least minimal statistical power. Kaplan–Meier estimators and log-rank tests (lifelines Python package) were used to compare survival distributions between aneuploidy groups for each aggregated or un-aggregated analysis. Multiple hypothesis testing correction utilized the false discovery rate Benjamini–Hochberg (BH) correction. Analyses with nominal *p*-values greater than 0.05 were considered not significant. Median survival times were derived from fitted Kaplan–Meier curves for each aneuploidy group, and significant results (*p* < 0.05) were plotted with 95% confidence intervals over a 60-month timeline. Results with greater median survival in loss lines can be found in Supplemental Table S1 and lower median survival in Supplemental Table S2.

### 2.4. Analysis of Whole Genome Duplication Versus Aneuploid Loss on Prognosis

We obtained TCGA genome-doubling calls from Genomic Data Commons (https://gdc.cancer.gov/about-data/publications/pancanatlas, accessed 9 June 2025) using calls from ABSOLUTE [45]. We binned values to a binary whole genome duplication (WGD) tag (≥1 doublings vs. no doublings). For each cancer type and aneuploidy event with ≥30 samples, we stratified patients into four groups: Euploid non-WGD, Loss non-WGD, Euploid WGD, and Loss WGD. We then compared survival between three predefined pairs (Euploid non-WGD vs. Loss non-WGD; Euploid WGD vs. Loss non-WGD; and Euploid WGD vs. Loss WGD) using Kaplan–Meier estimators and two-sided log-rank tests. Median survival times not reached (< 50% events) are denoted “NR”.

Further, to assess the relative prognostic impact of WGD versus aneuploidy across the pan-cancer cohort, we fitted a multivariable Cox model with progression-free survival as the outcome, including two binary covariates: WGD > 0 and presence of any chromosome arm loss. Model coefficients, hazard ratios, 95% confidence intervals, z-statistics, and two-sided *p*-values were reported.

### 2.5. Aneuploidy and Breast Cancer Subtype Prognosis Impact Analysis

To analyze the impact of different breast cancers (BRCAs), we evaluated BRCA cases with progression-free survival (in months) and disease-specific survival status. We binned survival events to a binary flag (where 1 = progression or death vs. 0 = censored). Progression-free survival was compared at a fixed landmark of t_0_ = 60 months within each breast cancer subtype (LumA, LumB, Her2, Basal, and Normal). For each subtype and each chromosome arm, samples were split into Loss vs. No Loss groups. Arms with fewer than 5 samples per group were omitted from analysis. Kaplan–Meier estimators were fitted separately to each group, and the survival probability at t_0_ was predicted. We recorded the difference

$${\Delta S=S}_{No\;Loss}\left(60\;mo\right)-S_{Loss}(60\;mo)$$

for each arm and subtype.

### 2.6. Broad PRISM ANOVA Analyses

Using the PRISM dataset, a one-way analysis of variance (ANOVA) was performed for each combination of either treatment (compound or MOA) and aneuploidy event (cancer types being aggregated together), or treatment, aneuploidy, and cancer type (un-aggregated). Multiple hypothesis testing correction utilized the false discovery rate Benjamini–Hochberg (BH) correction. Cell lines were excluded if lacking viability and/or aneuploidy information for the combination of treatment, chromosome arm, and cancer type during an un-aggregated analysis. ANOVA compared aneuploidy groups which were created from the samples meeting the inclusion requirements for each aggregated or un-aggregated analysis. Cell lines were assigned to either the “Loss” or “Other” aneuploidy group based on the aneuploidy status for that chromosome arm. An analysis was not performed if either aneuploidy group had fewer than 10 constituents to ensure at least minimal statistical power. This number was used as specific combinations of aneuploidy, treatment, and cancer type can be rare. Analyses with *p*-values greater than 0.05 were considered not significant. For significant comparisons (nominal *p* < 0.05), violin plots (Matplotlib, Seaborn) were generated to visualize the distribution of viability. Mean values in each group determined whether “Loss” lines exposed to the treatment were less or more viable than the “Other” lines. Using this, significant results were classified as either “Greater viability in loss lines” or “Lower viability in loss lines”.

### 2.7. TCGA MOA Mapping

The TCGA dataset lacked MOA assignments. To assign each applicable MOA to TCGA treatments, prompt engineering and large language models (LLMs) were leveraged. To map MOAs, the consensus was taken of 5 replicate responses from the API version of ChatGPT 4o mini (24 September 2024) for each TCGA treatment. For each possible pair of MOAs from the Broad dataset and treatments in the TCGA, the LLM was queried to return if “True” if treatment displayed that MOA and “False” if not. This was repeated five times for each pair, and the mode of the responses for the pair was used to assign if a PRISM MOA applied to a TCGA treatment. Useful mappings of these results were observed and manually validated. Undefined compounds and non-compounds (e.g., “radiation”) were removed. Treatments with unknown or unclear MOAs were not included in the analyses. MOAs of anti-tumor, anti-cancer, and anti-neoplastic activity were not included in the analyses as they applied to most compounds.

### 2.8. Overlapping Findings Between Datasets

The cancer types in the TCGA dataset are not identical to those in the Broad PRISM dataset. The Broad PRISM cell line name (e.g., “LN18_CENTRAL_NERVOUS_SYSTEM”) was used to derive the cancer type (e.g., CNS). Assigned mappings were as defined in Supplemental Table S3. Not all TCGA cancer types had an unambiguous match to a Broad cancer type and were not included. These mapped mutual cancer types were used to highlight MOAs with similar cytotoxic efficacy between the in vivo and in vitro datasets. While there are limitations in aligning cancer types in this manner, these limitations are inherent to cell line data with imperfect origin certainty and annotation. No singular compound showed identical selective cytotoxicity, likely due to the discussed disparate pharmacopeia of the two studies and the noted challenges with aligning the datasets.

### 2.9. Other Plots

JMP was used to generate heatmaps, bar charts, and dot plot used in this study using the graph builder feature. The Python package pyCircularize was used to generate chord plots from survival curve result data. Matplotlib and Seaborn were used to generate the explosion plot and other dot plots. The codes for the plots are included in the study’s associated GitHub repository.

### 2.10. Software Tools and Packages

Plotting and analyses were performed using JMP (18 Pro) and Python (3.11). The following python packages (and versions) were used: pandas (2.2.3), numpy (1.25.2), scipy (1.14.1), statsmodels (0.14.4), lifelines (0.29.0), matplotlib (3.9.2), pycirclize (1.7.1), seaborn (0.13.2), requests (2.32.3), and openai (1.43.0). Aneuploidy calculations for the cell lines utilized R (4.2.1), and RStudio (2024.09.1+394), including the packages data.table (1.15.4) and stringr (1.5.1).

## 3. Results

### 3.1. Derived Insights from Aneuploidy Treatment Landscape

**Figure 1:**
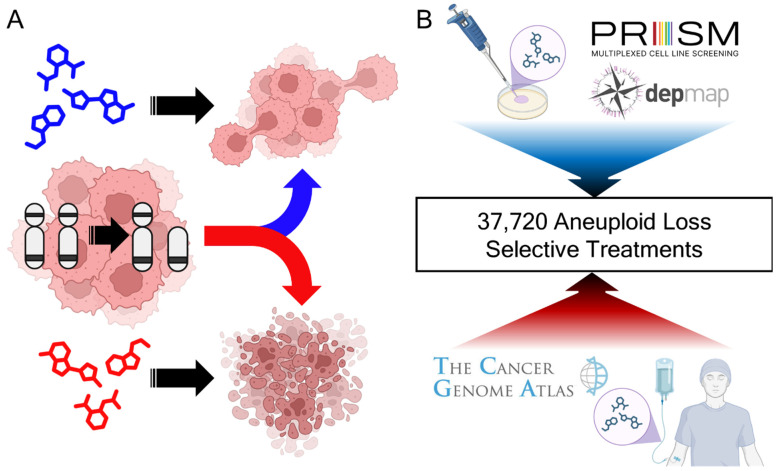
Extant cancer datasets are rich in aneuploidy-targeting treatment opportunities. (**A**) Cancer’s lose chromosome arms during their evolution. Aneuploid loss cancers are associated with variable cytotoxicity depending on the treatment; (**B**) 37,720 significant associations were identified with aneuploid loss, cancer type, treatment (single and aggregated by mechanism of action), and worsened or improved cytotoxicity.

Aneuploidy can influence cancer cell sensitivity to treatment. This concept is illustrated in Figure 1A, where variations in cytotoxic response are observed depending on the specific aneuploid loss state and chemotherapy applied. Our investigation of the TCGA and Broad PRISM datasets found 37,720 significant associations between chromosome arm loss and chemotherapy treatment with patient prognosis or cancer cell line viability (Figure 1B). In total, 22 treatments were observed with positive prognostic impact among the in vivo TCGA dataset and 46 with negative prognostic impact. Additionally, 17,946 associations between aneuploidy, cancer cell lines, and chemical treatments were observed in the in vitro Broad PRISM study. When aggregated by cancer type, 331 significant associations were shared. To identify the impact of pathways which may be shared between the TCGA and Broad studies, the mechanism of actions (MOA) of these compounds were examined, if known, resulting in 1338 TCGA MOA aneuploidy associations, 148 Broad MOA associations when aggregated by cancer type, and 16,189 Broad MOA associations when evaluated per cancer type. In total, 28 MOAs were observed to show similar cancer cytotoxicity between the TCGA and Broad PRISM study. The results can be found in Supplemental Tables S1–S9, which will be further described in the following analytical sections.

### 3.2. Aneuploid Losses Are Associated with Worse Outcomes on Current Chemotherapies

**Figure 2:**
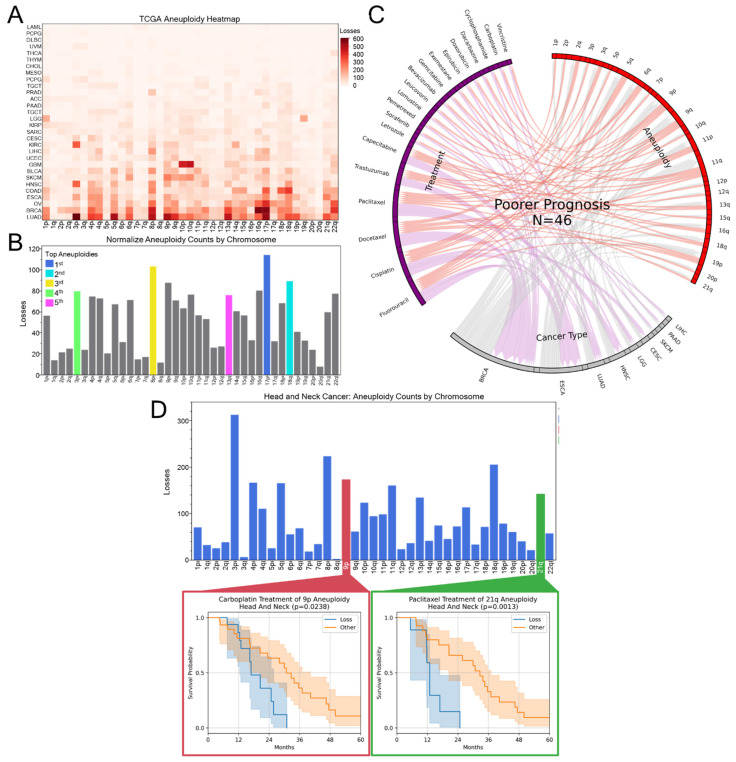
Cancer chromosome arm loss is often associated with poorer prognosis to treatments. (**A**) The count of TCGA aneuploid loss events by chromosome arm and cancer type. (**B**) The normalized counts of aneuploid losses, controlling for proportion of cancer types in the TCGA dataset. The first through fifth most common chromosome arm loss events are highlighted as follows: 17p (blue), 8p (yellow), 16q (cyan), 3p (purple), and 13q (violet). (**C**) Chord diagram showing the connection between treatments, cancer type, and aneuploidy that show worse 5-year prognosis (*p* < 0.05, N > 10 for both “Loss” and “Other” (not loss) aneuploidy statuses, as calculated by log rank test) in TCGA data as compared to patients without the aneuploid loss. (**D**) As a case example from Figure 2C., head and neck cancers with 9p aneuploid loss when treated with carboplatin and 21q aneuploid loss when treated with paclitaxel are associated with worse progression-free survival prognosis.

To evaluate how the clinical treatment of aneuploid cancers correlates with patient outcomes, aneuploidy events, treatments, and 5-year survival prognosis in the TCGA dataset were compared (Figure 2). Among cancer types, leukemia showed the least aneuploid loss events, consistent with previous studies (89), while non-small cell lung cancer (NSCLC/LUAD) had the greatest number (9468) (Figure 2A). A key for cancer-type abbreviations can be seen in Supplemental Table S3. Because some cancer types are overrepresented in the TCGA dataset, aneuploidy events were normalized to ensure that common events reflect biological prevalence rather than sampling bias. The most common aneuploidy event in normalized data was 17p (Figure 2B), putatively due to the loss of *TP53*-driven apoptosis regulation common in cancer. Infrequently lost chromosome arms included 3q, 8q, and 20q, which encode powerful oncogenes *PIK3CA*, *MYC*, and *SRC*, respectively, among others. Other common highlighted loss events such as 8p [46], 13q [47], 3p [48], and 16q [49] have been noted as being closely associated with a variety of cancers and poor prognoses. Aneuploidy was a stronger driver of chemotherapy resistance than ploidy. In a pan-cancer Cox model (see Supplemental Table S10), WGD (≥1 doublings vs. none) had no impact on progression-free survival (HR 1.002, 95% CI 0.92–1.09, *p* = 0.96), whereas any chromosome-arm loss doubled the hazard (HR 2.27, 95% CI 1.99–2.59, *p* ≈ 1.6 × 10⁻³⁴). As will be described, several of the highlighted common aneuploidy events are under- or overrepresented in our findings.

CIN and aneuploidy can facilitate chemotherapy resistance and cancer evolution [5,6,13,24]. Corroborating this, 46 treatments used in the TCGA were associated with worse prognoses in the presence of specific aneuploid alterations (Figure 2C). From these associations, several National Comprehensive Cancer Network (NCCN^®^)-recommended treatment options, including fluorouracil, appeared less effective or even deleterious in tumors harboring specific aneuploidies. Figure 2D illustrates two examples in head and neck cancer: patients with 9p aneuploidy treated with carboplatin and those with 21q aneuploidy treated with paclitaxel experienced poorer progression-free prognosis, consistent with a treatment interaction with the chromosome loss genotypes. Both treatments are recommended by the NCCN as possible chemoradiation options for head and neck cancers yet show worse prognosis in the outlined aneuploidies. The full list of significant negative prognosis associations can be found in Supplemental Table S2.

### 3.3. Positive Prognosis Associations from the TCGA

**Figure 3:**
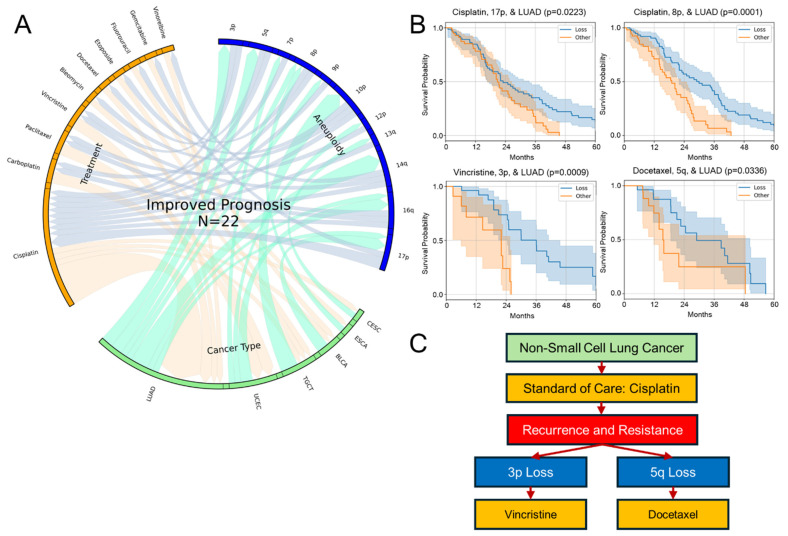
Specific aneuploid losses are associated with improved prognosis to select therapies. (**A**) Chord diagram showing the connection between treatments, cancer type, and chromosome arm loss shows improved 5-year prognosis (*p* < 0.05, N > 10 for both “Loss” and “Other” aneuploidy statuses, as calculated by the log rank test) in TCGA data as compared to patients without aneuploidy. (**B**) Example cases from Figure 3A of progression-free survival differences in patients with 3p and 5q aneuploid loss events in non-small cell lung cancer. (**C**) Using this information, a possible decision tree amenable to cancer institute retrospective analysis testing is proposed for precision treatment for non-small cell lung cancer based upon the tumor’s cancer aneuploidy.

Despite many studies indicating that general CIN is associated with poor prognosis, we sought specific positive survival associations for combinations of aneuploidy, cancer, and treatment. A total of 22 significant positive prognosis outcome associations were observed between these combinations of factors in the TCGA data (Figure 3A), notably fewer than negative associations, as predicted (46). Among the positive associations, the aneuploid event 16q was the most frequently represented but only the fifth most represented aneuploidy event among all cancer types in the TCGA dataset. Residing on 16q are many notable tumor suppressor genes including *LC3B* [50], *CYLD*, *CDH11*, *CDH1*, *ZFHX3*, *CREB*, *CTCP*, and all metallothioneins (*MTs*), which emerging research indicates are also tumor suppressors [51]. The most common cancer type yielding positively prognostic associations was NSCLC/LUAD (Figure 3A), possibly due to the high rate of aneuploid losses in these cancers observed in the TCGA dataset (Figure 2A). Interestingly however, breast cancer, the most common cancer type in the TCGA, was not proportionally represented in this positive prognosis analysis as it was with negative prognosis associations. This is in part due to its multiple genetic and hormonal subtypes (Supplementary Figure S1). It is noteworthy that some current chemotherapies target the aneuploid loss state and improve patient outcomes, despite the expectation of a worse prognosis due to higher CIN. This shows the potential for personalized treatment based on monoallelic loss profiles.

Variable treatment response to such standard-of-care chemotherapies including those in the TCGA dataset is at least in part driven by aneuploidy [37,52]. A potential high-impact connection in NSCLC was identified. Cisplatin showed the most representation among positively prognostic chemotherapies (Figure 3A,B), highlighting its potential effectiveness in targeting aneuploidy, as other studies have documented [12]. As such, treatment guidelines for NSCLC may begin with cisplatin, and then in the event of cancer, recurrence precision treatment may be considered. Aneuploid loss associations found here indicate that if a tumor contains a 3p loss (the most common aneuploidy in NSCLC), the best fit hypothesis to test is that it may be best treated with vincristine, while if it contains a 5q loss, it may best be treated with docetaxel (Figure 3B,C). Other potential clinical decision trees can be derived from the results presented here and those found in Supplemental Table S1 that may augment precision oncology. These differential prognoses may be considered to form the basis of chromosome loss testing and treatment assignment in future prospective clinical trials.

### 3.4. From Patient to Petri Dish: Cancer Cytotoxic Mechanisms of Action

**Figure 4:**
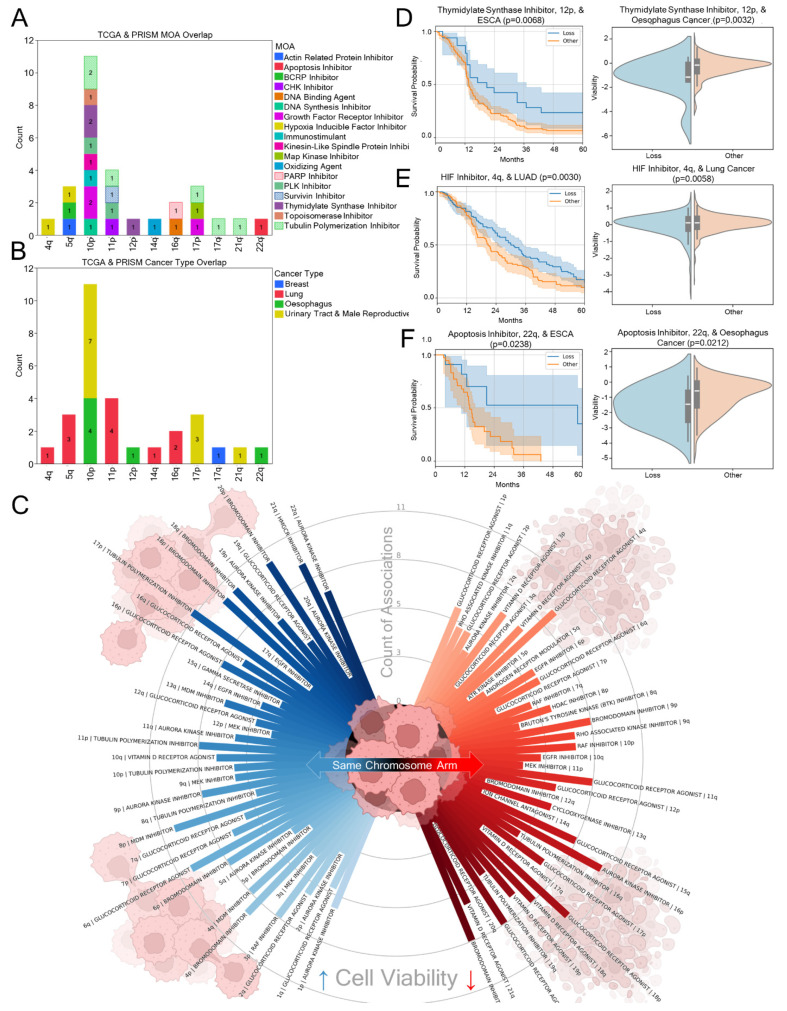
Mechanism of action study connects tumor drug selectivity to in vitro datasets. (**A**) The overlap between tumor TCGA data with improved 5-year progression-free survival and Broad PRISM reduced cell line viability data when treated with the same MOA. (**B**) Quantitation of overlap by cancer type. Note that some cancer types are underrepresented or not assessed due to cell line characterization limitations (see the Methods section). (**C**) An explosion plot of the all-cancers cell line PRISM data showing the most and least sensitizing MOAs by chromosome arm. The bar length is the count of significantly improved or reduced sensitivity by cancer types in the dataset. Identical chromosome arms are set radially opposed to one another. Blue lines show aneuploid losses which are less sensitive to an MOA, and red lines show those which are greater. (**D**–**F**) Cross-study comparison showing the progression-free survival (*p* < 0.05, N > 10 for both “Loss” and “Other” aneuploidy statuses, as calculated by log-rank test) and violin plot (*p* < 0.05, one-way ANOVA) comparisons between MOA treatment with (**E**) hypoxia inducible factor (HIF) in lung cancer, (**D**) thymidylate synthase inhibitors, and (**F**) apoptosis inhibitors in esophagogastric/oesophagus cancers for the TCGA and Broad PRISM samples.

Clinical data is highly influenced by variables like socioeconomic factors, lifestyle, and environmental exposure. PRISM datasets were used as a well-controlled study to identify small molecules, either canonical chemotherapies or drugs with a potential for repurposing, with aneuploid-loss-specific cytotoxicity without these confounders. As the TCGA and PRISM datasets had largely distinct pharmacopeias, mechanisms of action were assigned to treatments used in the TCGA data and unified by cancer type labels between the datasets (see methods). With this approach, 29 mechanisms of action were identified to both reduce cancer cell viability and improve patient 5-year survival prognosis significantly for cancer types bearing an aneuploidy (Supplemental Table S9). The most common interactions between the two analyses occurred on chromosome 10p (Figure 4A), primarily driven by urinary tract, male reproductive, and oesophageal cancers (Figure 4B). The overlap between the two datasets indicates pathways that may be targeted for treatment and provides cross validation for the cancer cytotoxicity findings observed in each dataset.

Evaluating how MOAs are associated with cancer cytotoxicity also highlights some unexpected classes of compounds with chemotherapeutic potential. In Figure 4C, among the statistically significant treatments that showed greater than median cytotoxicity across treatment dose concentrations (N = 7633), glucocorticoid receptor agonists (81), vitamin D receptor agonists (58), tubulin polymerization inhibitors (52), bromodomain inhibitors (49), and aurora kinase inhibitors (47) were the most common MOAs in the PRISM dataset when associated with cancer type and aneuploidy (Supplemental Tables S7 and S8). Despite glucocorticoid receptor agonists being only the fourteenth most common MOA among PRISM treatments, it was the most common among aneuploidy-related cytotoxicity. Interestingly, tubulin synthesis inhibitors are a common patient chemotherapy class and the 30th most common MOA in the cell line PRISM data, yet they showed strong association with greater median cell viability in 17p aneuploidy (Figure 4C). Given the range of activity and associations, three common significant associations are showcased between the PRISM and TCGA datasets. Thymidylate synthases inhibitors, such as fluorouracil, display enhanced cancer cytotoxicity with esophagogastric/oesophagus 12q aneuploid loss (Figure 4D). Chromosome arm 12p is the locus of the *KRAS* oncogene which, when lost, is associated with thymidylate synthase inhibitor sensitivity. Hypoxia inducible factor (HIF) inhibitors were associated with 4q aneuploidy cytotoxicity in lung cancer (Figure 4E). 4q hosts the gene *VEGFR2*, associated with angiogenesis and whose expression is regulated by HIFs [53]. A less clear relationship, but remarkably significant association, is that between esophagogastric/oesophagus cancer, 22q arm loss, and apoptosis inhibitor cytotoxicity (Figure 4F).

### 3.5. Putative Novel or Repurposed Arm-Loss Selective Chemotherapies

**Figure 5:**
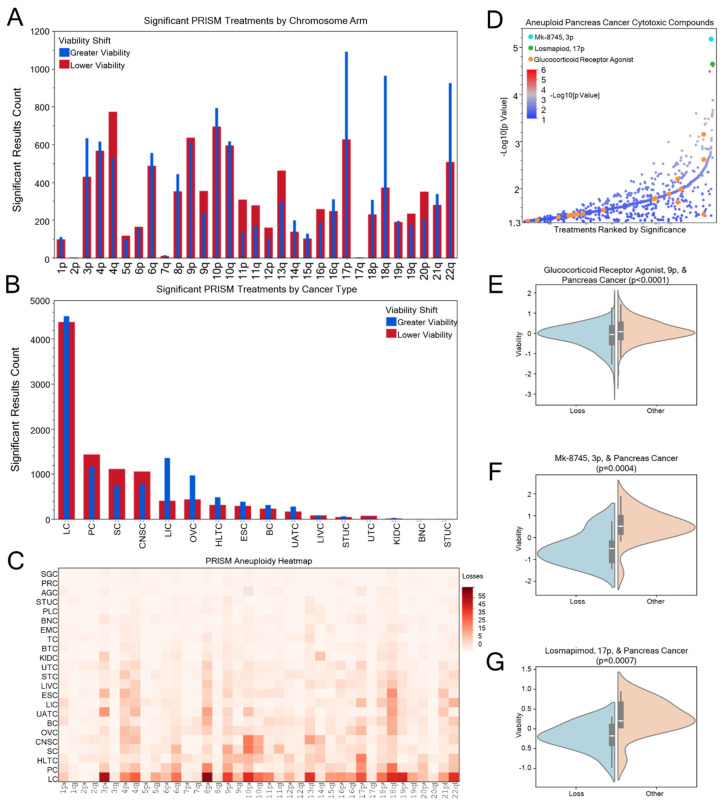
Identification of potentially novel arm-loss sensitizers, with a pancreatic focus. (**A**) Counts of significant treatment associations by chromosome arm in the Broad PRISM cell line dataset, with treatments associated with greater viability (less effective at killing cancer cells) shown in blue and lower viability (more effective) in red. (**B**) Counts of significant treatment associations by cancer type. (**C**) The count of aneuploid loss events calculated in the Broad PRISM dataset. (**D**) Significance of treatment associations in pancreatic cancer, with treatments ordered by ascending significance (−Log10[*p* Value]), with aurora kinase inhibitor Mk-8745 (cyan), MAPK inhibitor losmapimod (green), and glucocorticoid receptor agonists (orange) highlighted. Compounds with multiple significant aneuploidy associations are represented by individual points, with their mean value used to order their X-axis position. (**E**) Violin plots of glucocorticoid receptor agonist viability in pancreatic cancer cells with 9p aneuploidy, (**F**) Mk-8745 viability in pancreatic cancer cells with 3p aneuploidy, and (**G**) losmapimod viability in pancreatic cancer cells with 17p aneuploidy compared to other aneuploidy levels.

Cytotoxicity based on aneuploidy is known to be variable but also associated with CIN [13]. A total of 16,189 compound associations were identified between cancer cytotoxicity and specific aneuploid loss events (Figure 5A). For each chromosome arm (Figure 5A) and cancer type (Figure 5B), both positive and negative cytotoxic associations were identified. While these are a substantial number of significant findings, data type and structure limitations resulted in an underestimate of drug–aneuploidy associations. While many chromosome losses were observed (Figure 5C), specific arm losses were rare in the Broad PRISM cell lines, such as 17q, due to limitations in whole-arm copy-number analysis, as performed here (see methods). This is reflected in the absence of significant treatment associations for this smaller subset of chromosome arms. Despite pancreatic cancer being the sixth most aneuploid cancer type in human tumors, it showed the second most associations with treatment cytotoxicity (Figure 5B). In Figure 5D, a plot displays the significance of the substantial breadth of compounds which reduced pancreatic cancer cell viability preferentially in specific aneuploid losses as compared to other copy-number states. Glucocorticoid receptor agonists were highly represented among aneuploid loss-related cytotoxic compounds among many cancer types, including pancreatic (Figure 5D), particularly in 9p loss (Figure 5E). 3p loss exhibited selectivity with Mk-8745, an aurora kinase inhibitor, (Figure 5F) and 17p loss was associated with losmapimod sensitivity, a selective inhibitor of p38α and p38β MAPK (Figure 5G). Given the challenges of early detection, metastasis, and limited surgical options for pancreatic cancer [54], these findings may provide chemotherapy options that extend patient survival in a aneuploidy-based precision oncology trial setting. Supplemental Tables S5 and S8 highlight the results for each aneuploidy event among cancer types as a resource for future studies and clinical trials looking at compounds targeting specific aneuploid states.

### 3.6. Chemotherapies Selective for Multiple Concurrent Aneuploidies in Ovarian Cancer

**Figure 6:**
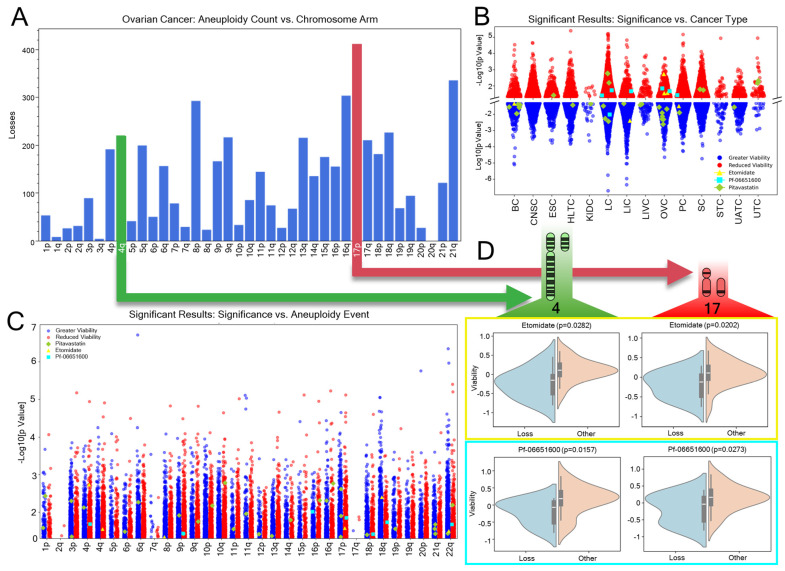
Notable arm-selective therapies for the treatment of ovarian cancer. (**A**) Counts of aneuploid loss from the TCGA dataset among ovarian tumors. 17p (red) and 4q (green) aneuploid loss events are the first and sixth most common events in ovarian cancer. (**B**) Dot plot of the significant associations between treatment, aneuploidy, and cancer type across the Broad dataset. On the positive Y-axis is the −log10 transformation of *p* values for treatments with significant cell viability reduction, and the negative Y axis is the log10 transformation of the *p* values. Red points are significant associations with reduced viability (more effective in cancer toxicity) when treated, and blue dots are those with higher viability (less effective). The treatments WWP2 repressor etomidate (yellow), JAK3 inhibitor Pf-06651600 (cyan), and cholesterol synthesis inhibitor pitavastatin (yellow green) are highlighted. (**C**) Significant associations by aneuploid loss are plotted with the same point highlighting coloration as (**B**). (**D**) Etomidate (yellow box) and Pf-06651600 (cyan box) treatments both significantly reduce cell viability in 17q (red) and 4p (green) chromosome arm loss as compared to other aneuploidy states (*p* < 0.05, as calculated by ANOVA).

Ovarian cancer genetics are dominated by aneuploidy. We posited that among these treatments, there should be a collection which is associated with reducing cell viability for multiple common aneuploid loss events of ovarian cancer (Figure 6A). A total of 369 treatments were associated with reduced cancer cell line viability with specific arm losses in ovarian cancers (Figure 6A,B). Two treatments, etomidate (a repressor of oncogenic E3 ubiquitin ligase WWP2 [55] and an anesthetic via GABA receptor agonism) and Pf-06651600 (ritlecitinib, a JAK3/TEC kinase inhibitor [56]), were associated with reduced cell viability in 17p (red in Figure 6A) and 4q (green in Figure 6A) aneuploid loss. These are the first and sixth most common ovarian aneuploidy events, respectively. In Figure 6D, we show that these drugs are preferentially cytotoxic to ovarian cancer cells with both aforementioned common 17p and 4q aneuploid losses. Pitavastatin was found to be generally associated with increased ovarian cell line viability across many aneuploidy types (Figure 6B,C). However, the opposite effect was found in Japanese patients taking pitavastatin, as compared to those on atorvastatin, having less carcinogenesis [57]. As expected, this may be due to different effects when studied in vivo or in vitro, as will be later discussed. Other limitations in the data include few significant treatment associations with 2q, 7q, and 17q loss events. Similarly, some chromosome arms, such as 1q, are absent from Figure 6C due to lacking sufficient arm loss calls to perform differential analysis. Despite these limitations, the significant associations identified in our study provide valuable hypotheses for future precision oncology research focused on aneuploid losses in ovarian cancer. In particular, the unique two-hit aneuploid loss events, which result in dual sensitization to therapy, warrant further investigation.

## 4. Discussion

Our findings highlight that aneuploidy, a hallmark of cancer, is not merely a byproduct of genomic instability but is indeed a critical factor influencing how tumors respond to various therapies. By leveraging both clinical (TCGA) and in vitro (PRISM) datasets, pathways and compounds were identified that may refine treatment decisions in the era of precision oncology. As we and others have observed, aneuploidy and CIN further complicate successful treatment, often worsen patient prognosis, and often provide chemotherapy resistance [8,12,13,15,25,32,47,48,49]. Cancer is challenging to treat with cytotoxic chemotherapy as treatments are also toxic to the patient [58,59]. However, the general CIN state and specific aneuploidies of cancer incur specific vulnerabilities to be exploited for treatment, preferentially targeting the disease over the host [2,58]. Over 35,000 possible beneficial and deleterious associations were identified herein between aneuploid losses and treatment. The monoallelic state influences and is influenced by compounds that take advantage of biology changed by aneuploidy.

Treatment approaches recommended by organizations such as the National Comprehensive Cancer Network have evolved from a uniform approach to truly personalized medicine, taking into account various biomarkers, particularly in hematologic cancers. As new evidence emerges, treatment guidelines continue to shift. In this study, compounds which were capable of targeting the monoallelic state of cancer cells were identified to potentially advance precision oncology in solid tumors. To understand the current state of treatment, our analysis first highlighted treatments linked to poorer prognosis. For example, paclitaxel has shown greater effectiveness in patients with more chromosomal abnormalities in general [60]. However, specific aneuploid chromosome arms in individual cancer types clearly influence response. While 21q amplification can confer resistance to paclitaxel in epithelial cancer cells [61], no information on the effects of 21q loss was found in the literature. Here, a negative association was discovered between 21q aneuploidy and patient prognosis when treated with paclitaxel in head and neck cancer. In nasopharyngeal cancers, 21q aneuploidy is associated with changes in mitosis regulation and microtubule polymerization, pathways targeted by paclitaxel. Many chemotherapies, including paclitaxel, show reduced effectiveness against highly aneuploid or polyploid cancers, partly due to these cells’ slower progression through G1 and S phases [12]. Because standard-of-care chemotherapies often target cell cycle stages, alterations in commonly found in cancer’s chromosome content and regulation can diminish chemotherapeutic efficacy. This variability in response demonstrates the necessity of integrating aneuploidy profiling into clinical decision-making. Supporting this approach, we examined whether other studies using TCGA data have linked gene–drug sensitivity to the aneuploidy–drug sensitivities we observed. In cervical cancer, the TCGA’s 2017 integrated genomic characterization study reported LOF mutations in RB1 (13q)-sensitize tumors to cisplatin through altered PI3K/AKT signaling [62]. We observed similar results with cisplatin therapy and improved survival prognosis in 13q aneuploidy. Ultimately, however, not all patients will benefit from the same therapy, even within the same tumor type, due to underlying genomic complexities. This is currently appreciated for the single-nucleotide variant and homozygous loss drivers, but aneuploidy is currently undervalued in prognostic assessments.

Mitotic catastrophe was a common theme in our aneuploid-targeting results. Aneuploidy arises from dysregulated DNA segregation and repair, coupled with insufficient mechanisms to recognize such genomic distress. Docetaxel, a positively prognostic example, results in double-strand breaks and failed chromosome segregation, increasing genomic instability. Once exploited, this existing weakness in aneuploid cancer cells leads to mitotic catastrophe. However, docetaxel can also promote further aneuploidy and polyploidy, potentially fueling cancer evolution and resistance [63]. Similarly, vincristine, an anti-microtubule agent that inhibits mitosis, can induce polyploidy and thus foster treatment resistance [64]. Targeting mitosis in aneuploid cells can yield positive or negative outcomes: it may increase genomic instability to lethal levels or drive further aneuploidy changes that enhance resistance. As stated, this resistance partly stems from slower replication rates in highly aneuploid and polyploid cells [12]. Similarly, looking at the larger pool of drugs in the in vitro results, some highlighted compounds from Figure 5, such as the aurora kinase A inhibitor Mk-8745, can also further escalate aneuploidy and polyploidy, leading to mitotic catastrophe and cell death [65]. Mk-8745, known to induce tetraploidy, may also consequently return a semblance of euploid gene expression and reactivate haploinsufficient apoptotic pathways [66]. Other compounds, like losmapimod, a p38α/β MAPK inhibitor, may induce direct apoptosis in cells acquiring an aneuploid state via p38-mediated pathways [67]. Furthermore, p38 inhibition can exacerbate genomic instability and aneuploidy, again pushing cancer cells toward mitotic catastrophe [68,69].

Given the high prevalence of aneuploidy in cancer, compounds that target multiple common aneuploidies within a cancer type may offer more durable treatment options. Further, these compounds may treat the unique CIN state common among a cancer type. Examining compounds that target two of the most common ovarian cancer aneuploidies concurrently (4q and 17p), Pf-06651600/ritlecitinib was identified as a potentially effective therapeutic, which disrupts the JAK3-STAT pathway (Figure 6). Similar JAK3 inhibitors induce apoptosis in natural killer/T-cell lymphoma [70]. Pf-06651600 also inhibits five TEC kinases, including Bruton’s tyrosine kinase, known to support cancer cell survival and proliferation [71,72]. TEC kinase phosphorylation regulates PLK4, which influences liver cancer metastasis and centriole duplication. Improper PLK4 activity can lead to lethal chromosomal segregation issues in already dysregulated cancer cells [73,74]. Another dual aneuploidy-targeting compound, etomidate, reduces the expression of the oncogenic protein WWP2, thereby limiting cell proliferation and inducing apoptosis in non-small cell lung cancer [55]. The overexpression of WWP2 correlates with poor prognosis and proliferation in gastric [75], liver [76,77], lung adenocarcinoma [78], and ovarian cancers [79]. WWP2 interacts with SMAD proteins within the TGFβ pathway, which governs cell proliferation, migration, differentiation, and apoptosis [80,81]. While TGFβ increases aneuploidy rates in cancer cells, it suppresses proliferation in normal epithelial cells [82,83]. One mechanistic hypothesis resulting from this study is that etomidate represses WWP2 and TGFβ signaling in cancer cells reliant on these proliferative pathways. Without TGFβ’s influence, the induced aneuploidies undermine the cancer cells’ adaptations to a high-TGFβ environment.

Drug MOAs yielded aneuploid-specific cytotoxicity consistent with the previous literature and an understanding of targets encoded on lost chromosomes. Each MOA comprises various drugs, and some may be preferred clinically for their unique properties, such as blood–brain barrier penetration or targeted organ localization. Three MOAs are highlighted that show both clinical and in vitro cytotoxicity against aneuploid cancers: thymidylate synthase inhibitors, hypoxia-inducible factor inhibitors, and apoptosis inhibitors. Prior work demonstrates that 5-fluorouracil, a thymidylate synthase inhibitor, sensitizes NSCLC *KRAS* mutants to apoptosis via the TRAIL-mediated pathway [34]. The loss of 12p, a locus that includes *KRAS*, may reduce wild-type *KRAS* expression and thus enhance sensitivity to thymidylate synthase inhibitors. Loss of heterozygosity in *KRAS* is associated with improved prognosis for patients with early-stage lung adenocarcinoma [84]. Similarly, hypoxia-inducible factor inhibitors can be effective against 4q aneuploidy in lung cancer. *VEGFR2* (*KDR*), located on 4q12, is modulated by hypoxia-inducible factor-1α. The downregulation of *VEGFR2* induces apoptosis in breast cancer cells [85], while its upregulation drives angiogenesis, proliferation, and metastasis in colon cancer [86]. Mono- and bi-allelic loss of *VEGFR2* represses mouse tumor growth equally, illustrating its haploinsufficient role in cancer progression [87]. Thus, 4q aneuploidy may reduce *VEGFR2* expression, and concurrently inhibiting hypoxia-inducible factor-1α further diminishes cancer viability.

While some therapies target specific gene losses on the affected chromosome, others likely interact with multiple genes that exert moderate oncogenic influence and/or contribute to an aberrant CIN state. As observed here, apoptosis inhibitors can be paradoxically effective in killing esophageal cancers with 22q aneuploidy. Chromosome 22q harbors multiple tumor suppressors and apoptosis-related genes (e.g., *APOBEC3B*, *CHEK2*, *CLTCL1*, *EP300*, *LZTR1*, *MKL1*, *MYH9*, *NF2*, *ATF4*, *MAPK1*, *MAPK12*, *CARD10*, and *XRCC6*). Loss of these factors can cause accumulation of genetic defects. For example, loss of *CHEK2* allows for the bypass of DNA damage checkpoints, facilitating unchecked replication and aneuploidy [88,89]. Another 22q resident gene, *CARD10*, is a caspase regulator involved in apoptosis and the NF-κB signaling pathway [90,91]. The germline loss of 22q11.2 results in DiGeorge syndrome and is associated with increased risk for many types of cancer [92]. In cells with 22q aneuploidy, treatment with apoptosis inhibitors may further allow for the accumulation of metabolic or genomic defects in cancer cells lacking repair mechanisms, leading to non-apoptotic cell death (e.g., mitotic catastrophe and necrosis) [7]. These findings underscore how aneuploidy serves as a critical determinant of therapeutic effectiveness, even for well-established mechanisms of action.

Glucocorticoid receptor (GR) agonists emerged as a prominent, and unexpected, predicted effective therapy discovered in the well-controlled cell-line data. Reduced GR expression in cell lines correlates with changes in aneuploidy levels during passage, and GR haploinsufficiency increases tumor incidence in mice [93]. GR localization at the mitotic spindle is essential for normal mitotic progression. Low GR expression is linked to multiple human malignancies, including liver, lung, prostate, colon, and breast cancers. GR activation can repress *MYC* and other cell cycle genes, slowing proliferation [94]. GR also increases BIM and decreases BCL2 expression, pushing lymphocytic leukemia cells toward apoptosis [94]. Notably, the overexpression of *NR3C1*, the sole gene encoding GR, promotes proliferation and migration in renal clear cell carcinoma, where it is often duplicated and rarely repressed in TCGA samples [95]. High GR expression is associated with poor prognosis in estrogen receptor-negative breast cancer, inducing epithelial to mesenchymal transitions, but improved prognosis in estrogen receptor-positive cancers [96]. These findings suggest that GR agonists may induce changes in genomic segregation driving cancer evolution but may also force aneuploid cells into mitotic death, much like docetaxel. GR agonists, such as dexamethasone and prednisolone, are standard treatments for childhood leukemias, typically effective in combination therapies (e.g., with vincristine), though alone they often provide only temporary remission or symptom relief [94]. Interestingly, our findings reveal the cytotoxic effects of GR agonists in cell lines specifically associated with aneuploidy, independent of previously described mechanisms involving the tumor microenvironment, immune modulation, or systemic hormonal signaling. These concepts emphasize that the impact of GR agonists in cancer may depend on the genomic context, switching between pro-cancer and anti-cancer effects, just as GR can toggle between pro- and anti-inflammatory responses depending on cellular context [97,98]. Given the significant number of cytotoxic associations observed, the benefits of further research into the clinical applications of GR agonists are warranted.

Chromosome arm losses, as we have shown, are associated with differences in therapeutic efficacy. The mechanisms by which aneuploidy arises, whether through chromosomal instability (CIN) or by triggering further CIN, drive cancer evolution. This ultimately influences therapy resistance or susceptibility. CIN not only promotes the accumulation of aneuploidy but also arises because of genome architecture changes during tumor progression [6]. Likewise, studies of WGD show that WGD confers therapy resistance by buffering deleterious chromosomal losses and fueling additional CIN, thereby increasing karyotypic heterogeneity and affecting treatment response [99,100]. In our study, all cancer types harbored both diploid and tetraploid (WGD) tumors (Supplemental Table S10). Although our analysis focused on aneuploid losses relative to other ploidy states, future work investigating the effects of WGD and CIN on therapy outcomes is warranted. Our findings and these recent reports collectively emphasize the importance of considering WGD and CIN as central players in the biology and clinical consequences of aneuploidy.

The limitations of our study prevent a fully comprehensive evaluation of all aneuploidy–drug interactions. In the PRISM analysis, we were able to focus exclusively on cell-intrinsic biology, which constrained our ability to observe tumor microenvironmental factors. Some treatments may re-sensitize the immune system by modulating haploinsufficient pathways masking cancer cells from immune detection. *PTEN* deficiency, for example, can create immunosuppressive tumor microenvironments by impairing interferon responses, altering secretion of immunosuppressive compounds, and increasing neoantigen production [32]. Certain treatments might be more effective in vivo by interrupting angiogenesis, reshaping the stroma, or affecting other tissue-level factors [101,102,103]. Like canonical mutations, the heterogeneity of tumor cells, each with varying aneuploidy, poses a risk of resistance when targeting a vulnerability that can evolve [104]. Integrating single cell sequencing and spatial transcriptomics may elucidate how aneuploid heterogeneity within tumors influences therapeutic outcomes. While in vitro findings often do not translate directly to clinical outcomes, some aneuploidy-targeting treatments may indeed prove more potent in the complex in vivo environment. However, identifying effective aneuploidy-associated therapies from patient data remains challenging. Novel treatments, including those exhibiting aneuploidy-specific cytotoxicity, are inconsistently documented even in harmonized cancer databases. Additionally, aneuploidy and copy number alterations are rarely reported in early-phase clinical trials. Future research should aim to confirm our findings in animal models and early-phase clinical trials that incorporate aneuploidy assessments. Addressing the current shortcomings in cancer aneuploidy reporting, both in clinical practice and trials, is essential as oncology evolves from individual-biomarker-based treatment decisions toward a more comprehensive, genomics-driven approach.

## 5. Conclusions

In summary, many standard-of-care treatments correlated with poorer prognosis in aneuploid cancers, while fewer aneuploid states correlated with improved outcomes. Encouragingly, multiple treatments exhibit cytotoxic interactions with specific cancer types and aneuploidies. Although only a few examples are highlighted here, comprehensive lists are provided in Supplemental Tables S1–S9. Several mechanisms underlying aneuploid cancer cell vulnerability to chemotherapy are proposed: amplifying genetic or metabolic instability beyond viability thresholds, restoring haploinsufficient apoptosis regulators, driving gene expression to lethal imbalances, and disrupting haploinsufficiency-induced chemotherapy resistance pathways. This study will serve as a resource for future research aimed at unraveling why certain treatments are more successful in specific cell lines, patients, and aneuploidies and, ultimately, improving precision oncology-based care.

## Data Availability

The supporting datasets are supplied in the Supplementary Tables. The code used to prepare and analyze the datasets can be found at https://github.com/jrdelaney/Disharoon_Delaney_Aneuploid_Selectivity_2025/ as of 13 June 2025.

## Supplementary Materials

The following supporting information can be downloaded at https://www.mdpi.com/article/10.3390/genes16060708/s1, Supplemental Figure S1: Overall survival changes with aneuploidy by BRCA subtype. Supplemental Table S1: TCGA positive survival prognosis associations. Supplemental Table S2: TCGA negative survival prognosis associations. Table S3: Cancer type abbreviations. Supplemental Table S4: Broad drug viability associations by cancer type. Supplemental Table S5: Broad drug viability associations aggregated. Supplemental Table S6: TCGA MOA survival prognosis associations. Supplemental Table S7: Broad MOA viability associations aggregated. Supplemental Table S8: Broad MOA viability associations by cancer type. Supplemental Table S9: Common effects between TCGA and Broad MOAs. Supplemental Table S10: CoxPH PFS analysis of WGD and aneuploidy.

## Abbreviations

The TCGA standard abbreviations (see https://gdc.cancer.gov/resources-tcga-users/tcga-code-tables/tcga-study-abbreviations, accessed on 7 October 2024) and PRISM abbreviations are provided in Supplementary Table S3. The following abbreviations are used in this manuscript:

ANOVA	Analysis of variance
BH	Benjamini–Hochberg
CIN	Chromosome instability
CNA	Copy number alteration
GR	Glucocorticoid receptor
LLM	Large language models
MDPI	Multidisciplinary Digital Publishing Institute
MOA	Mechanism of action
MT	Metallothionein
NCCN	National Comprehensive Cancer Network
NR	Not reached
NSCLC	Non-small cell lung cancer
PRISM	Profiling Relative Inhibition Simultaneously in Mixtures
TCGA	The Cancer Genome Atlas
WGD	Whole-genome duplication
