# Supplementary figures and images for: Half the Chromosome It Used to Be: Identifying Cancer Treatments Targeting Aneuploid Losses
Source: Genes (Basel). 2025 Jun 14;16(6):708. doi: 10.3390/genes16060708 (PMC12192454; doi:10.3390/genes16060708)

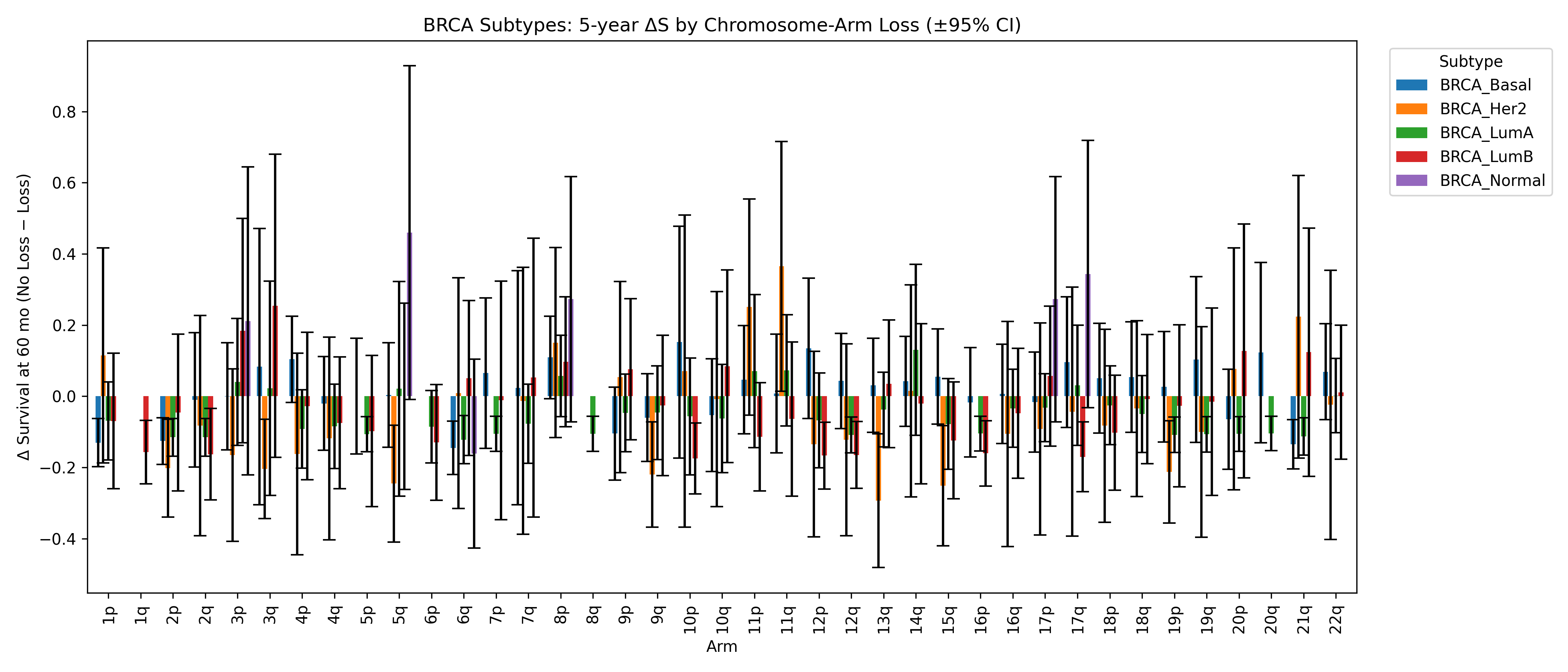

Supplement: Supplementary file 1 [file genes-16-00708-s001.zip › Supplemental Figure S1.png]
